# Relationships Among COVID-19-Related Service Uptake, HIV Status, Drug Use, and COVID-19 Antibody Status Among HIV Testing Intervention Participants in KwaZulu-Natal, South Africa

**DOI:** 10.3390/ijerph21111411

**Published:** 2024-10-25

**Authors:** Leslie D. Williams, Phumlani Memela, Alastair van Heerden, Samuel R. Friedman, Phillip Joseph, Buyisile Chibi

**Affiliations:** 1 Division of Community Health Sciences, School of Public Health, University of Illinois Chicago, Chicago, IL 60612, USA; 2 Centre for Community Based Research, Human Sciences Research Council, Sweetwaters 3201, South Africaavanheerden@hsrc.ac.za (A.v.H.); pjoseph@hsrc.ac.za (P.J.);; 3 Developmental Pathways for Health Research Unit, Department of Paediatrics, School of Clinical Medicine, Faculty of Health Sciences, University of the Witwatersrand, Johannesburg 2017, South Africa; 4 Department of Population Health, New York University Grossman School of Medicine, New York, NY 10016, USA; samuel.friedman@nyulangone.org

**Keywords:** COVID-19 service uptake, COVID-19 testing uptake, COVID-19 vaccine uptake, HIV status, undiagnosed HIV, hard drug use, COVID-19 IgG antibody status

## Abstract

People living with HIV (PLWH) and people who use drugs are vulnerable populations who may face barriers to accessing health services and may have irregularities in immune function. People with undiagnosed HIV infection may be particularly likely to have compromised immune function. However, research about whether/how HIV status is related to COVID-19-related health outcomes has been equivocal, and research on the predictors of COVID-19-related health service access/uptake has been limited in Sub-Saharan African settings. Among 470 participants of a peer-recruitment-based HIV-testing intervention in KwaZulu-Natal, we examined whether HIV status and/or hard drug use were associated with uptake of COVID-19 testing and vaccination, and whether they moderated the relationship between COVID-19 vaccination status and COVID-19 IgG antibody status. Women were significantly more likely than men to report testing for COVID-19 (OR = 1.84; *p* = 0.002) and being vaccinated (OR = 1.79; *p* = 0.002). Neither HIV status nor drug use was associated with likelihood of getting tested or vaccinated. Vaccinated participants (90% of whom obtained vaccines more than 6 months before the study) were significantly more likely to test positive for COVID-19 IgG antibodies (OR = 6.86; *p* < 0.0005). This relationship held true for subgroups of PLWH and participants with previously undiagnosed/uncontrolled HIV infection, and was not moderated by HIV status or hard drug use. These findings may suggest that both people who use drugs and PLWH were served as well as other people by KwaZulu-Natal’s COVID-19 response. However, gender-based disparities in COVID-19 service uptake suggest that special care should be taken during future COVID-19 outbreaks or other new epidemics to improve access to related healthcare services among men in this region.

## 1. Introduction

Vaccination is an important part of COVID-19 prevention and can reduce the likelihood of severe disease when infection occurs. Given the importance of adequate immune functioning to COVID-19 outcomes, COVID-19 testing and vaccination could theoretically be of particular importance for people living with HIV (PLWH), particularly if they do not have a suppressed HIV viral load. However, research on the relationship between HIV infection and COVID-19 outcomes has been quite equivocal, especially regarding risk of mortality [1]. Some studies that have found no relationship between HIV infection and severity or outcomes of COVID-19 infection have relied on samples predominately composed of people already on antiretroviral therapy (ART) or have not measured ART status [2] or have compared only relative severity of COVID-19 outcomes among PLWH and non-PLWH who were hospitalized for COVID-19 [3]. Most meta-analyses and systematic reviews, on the other hand, have suggested increased risk for at least some COVID-19 outcomes (e.g., more severe infection) among PLWH [1,4,5].

Research on COVID-19 vaccine antibody responses among PLWH ***receiving ART*** have reported high antibody seroconversion rates after vaccination [6], and similar antibody responses between PLWH receiving ART and comparison groups of HIV-negative participants [7], except among individuals with low CD4^+^ T cell counts, for whom antibody responses have been found to be poorer, even among individuals who were receiving ART at the time of vaccination [8]. However, there remains a great deal yet to be understood about COVID-19 health outcomes and vaccine responses among people who have uncontrolled (i.e., undiagnosed and/or untreated) HIV. Increasing this understanding is important, and in the meantime, it is important to ensure the best possible access to COVID-19 testing and vaccination in geographic settings such as South Africa that, due to high HIV prevalence and incidence rates, are likely to have large numbers of people with undiagnosed and/or untreated HIV. It is also important to understand access to and uptake of COVID-19 testing and vaccination among diagnosed PLWH in such settings, since research has found that HIV-related stigma can act as a barrier to accessing healthcare of various kinds among PLWH in South Africa [9,10,11,12]. However, little is known about whether access to COVID-19-related services (e.g., testing and vaccination) has varied as a function of HIV status among South Africans.

People who use drugs (PWUD) are another population whose COVID-19 service access is not adequately understood in high-HIV-prevalence settings like South Africa. Drug use is associated with higher risk for HIV, due to greater likelihood of increased sexual risk-taking [13,14,15,16] and sex work [17,18] or transactional sex [19] among people who use drugs, and due to HIV transmission risk conferred by drug injection. People who use drugs have also been found to be more likely—in many settings globally—to have lower or more inconsistent access to healthcare, in part due to stigma [20,21,22,23,24]. This lower access to health services, generally, may mean that this population is at risk of being underserved by COVID-19-related health services such as testing and vaccination. Low access to healthcare among PWUD may also increase the likelihood that PWUD in a high HIV prevalence setting like South Africa are more likely to be HIV-positive-unaware (i.e., to have uncontrolled/untreated HIV infection). Additionally, people who use drugs have been found to have heightened immune activation [25,26,27], which can lead to increased inflammation and progression of infections [28,29,30]. These factors could make COVID-19 testing and vaccination particularly important for people who use drugs. However, to our knowledge, there is currently no published scientific evidence on COVID-19-related health service access among people who use drugs in South Africa.

We report on COVID-19-related service uptake and COVID-19 immunoglobulin G (IgG) antibody status among a sample of participants from an HIV testing intervention in 2022–2023, more than 2 years into the COVID-19 pandemic. Since the intervention study (a) aimed to recruit people who use drugs and (b) diagnosed many participants with HIV at the time of data collection (i.e., sampled many participants with undiagnosed/uncontrolled HIV at the time of data collection), use of this sample provides a unique opportunity to conduct descriptive and exploratory analysis of COVID-19 service uptake and antibody status among both of these potentially vulnerable groups. Among this sample, we addressed the following research questions:

1. Are key sociodemographic characteristics (e.g., gender and/or age) associated with COVID-19-related service uptake (i.e., vaccination and testing)?
2. Do HIV status or drug use predict COVID-19-related service uptake?
3. Do drug use and/or HIV status (including having uncontrolled/previously undiagnosed HIV) moderate the relationship between COVID-19 vaccine status and COVID-19 IgG antibody status?

## 2. Methods

### 2.1. Sample and Procedures

The present sample includes all participants (N = 470) of a study to compare the ability of two network-based strategies to recruit men to HIV testing, to diagnose people who were HIV-positive-unaware and link them to treatment, and to reduce HIV-related stigma. Between July 2022 and March 2023, initial (i.e., seed) participants were recruited from two clinics and one drug treatment center in Msunduzi municipality in KwaZulu-Natal province, South Africa, and had to have been recently diagnosed with HIV (within the last two months) to be eligible to be seed participants. We asked each seed participant (N = 110) to recruit other people they knew to be tested for HIV and to join the study, using different recruitment instructions for each of the two study arms to which seeds were randomized: (1) recruitment of direct risk partners or (2) expanded network recruitment (in which we asked participants to recruit anyone they knew who they believed might benefit from HIV testing). All network members recruited directly by a seed participant were tested for HIV and were also asked to recruit others to join the study, according to the same recruitment strategy by which they were recruited. These additional network members were also tested for HIV. All network members received HIV testing and counselling, and all participants received care referrals and help with care linkage. This study was approved by Institutional Review Boards at the University of Illinois Chicago in Chicago, United States (protocol # 2020-0997) and at the Human Sciences Research Council in Pietermaritzburg, South Africa (protocol # REC 3/20/05/20). All participants completed a process of written informed consent. Full details on the procedures for this study have been published in Williams et al., 2024 [31].

### 2.2. Measures

Key sociodemographic characteristics assessed included participant gender and age, which were measured using participant self-report. Gender is operationalized as a binary variable, since all participants reported being either cis-gender men or cis-gender women. Age is operationalized as a binary variable, using the median age as a cutoff, categorizing participants as either aged 18–31 or as 32 years and older.

COVID-19 testing uptake was measured as a binary variable in which participants self-reported on whether or not they had been tested for COVID-19 by a nurse or healthcare provider since the start of the pandemic in 2020. COVID-19 vaccination uptake was also measured as a binary variable reflecting participant self-report on whether or not they had been vaccinated for COVID-19. Participants were also asked how many vaccine shots/doses they had received and how long ago their last shot/dose was received. These additional details were used for descriptive purposes only. The analyses for the present study that test for associations with other variables use the binary variable indicating whether or not, based on participant self-report, participants had received at least one dose of COVID-19 vaccine at any time preceding study data collection. COVID-19 IgG antibody status was measured using an in vitro immunochromatographic COVID-19 IgG antibody rapid assay by Orient Gene [32], performed using whole blood samples.

HIV status was measured both as a binary variable (HIV-positive/HIV-negative) and as a categorical variable that included three categories: HIV-negative, HIV-positive and already aware at baseline, and HIV-positive-unaware at baseline (i.e., newly diagnosed by the present study). Participants were considered to be HIV-positive-unaware at baseline (i.e., newly diagnosed with HIV or NDH) if they tested positive for HIV during study-administered testing and counselling and indicated during their pre-testing interview that they had never tested positive for HIV before. Hard drug use was measured as a binary variable indicating whether participants self-reported using any drug other than alcohol or marijuana for recreational purposes in the last six months. Specifically, after having been asked about the frequency of their alcohol and marijuana use, participants were then asked to report on whether they used each of the following types of drugs in the last six months: nyaope, amphetamines, methamphetamines, cathinone, cocaine, crack, steroids, opium, heroin, other narcotics such as prescription opioids, or psychedelics such as lysergic acid diethylamide (LSD) or phencyclidine (PCP) or methylenedioxymethamphetamine (MDMA). Several slang names for each of these drug types were also provided to participants along with the names listed above. Participants were also given the option to report on other types of drugs about which we did not specifically ask. To reduce potential participant discomfort and/or social desirability bias, participants were asked to enter their responses to each question about substance use privately on the mobile devices used to capture data and to toggle to the next question before handing the device back to the interviewer, so that the interviewer was not aware of their responses to these questions.

### 2.3. Analyses

Analyses were conducted using SPSS version 26. First, descriptive statistics were computed. Then, bivariate relationships exploring the relationships of gender, age, hard drug use, HIV status, and newly diagnosed with HIV (NDH) status to COVID-19-related service uptake (i.e., to COVID-19 vaccine status and COVID-19 testing uptake) were tested using both Chi-square tests and binary logistic regression analyses (in order to compute 95% confidence intervals). As a sensitivity analysis only (since the present study is exploratory and the sample size within each cell is relatively small), we also tested two adjusted logistic regression models that included HIV status (PLWH or not), hard drug use, gender, age, employment, and completion of high school as independent variables and either COVID-19 vaccination uptake or COVID-19 testing uptake as outcomes. Similarly, we tested the relationship between COVID-19 vaccine status and COVID-19 IgG antibody status using both Chi-square and binary logistic regression analyses. Then, to test whether hard drug use, HIV status, or NDH status moderate the relationship between COVID-19 vaccine status and COVID-19 IgG antibody status, three separate adjusted logistic regression models were conducted that each included one interaction effect (and the two main effects comprising the interaction effect) as independent variables and COVID-19 IgG antibody status as the dependent variable. Then, as a sensitivity analysis only (again given the small sample sizes for specific subgroups), we repeated these three adjusted logistic regression models with sociodemographic characteristics added as covariates.

## 3. Results

### 3.1. Descriptive Statistics

Descriptive statistics for key variables are presented in Table 1. Almost 11% of participants reported using hard drugs in the last six months. Nyaope was by far the most commonly used specific drug, with 48 participants (10.2%) reporting using it at least “a few times” in the last six months. The sample included 53 participants (11.3%) who were HIV-positive-unaware (i.e., who were diagnosed with HIV by the present study), 180 participants (38.3%) who were HIV-positive-aware before joining the study, and 237 participants (50.4%) who were HIV-negative.

With regard to uptake of COVID-19-related services, 52.6% of participants reported having received at least one COVID-19 antigen test from a nurse or healthcare provider prior to their participation in the study. About 47.4% of participants reported having received at least one dose of COVID-19 vaccine. Of those, 85.2% reported that they received their most recent vaccine dose over six months prior to study participation.

### 3.2. Associations between Sociodemographic Characteristics and COVID-19 Service Uptake

Table 2 presents Chi-square test results and logistic regression results for analyses examining associations between sociodemographic characteristics and COVID-19 service uptake. Gender was significantly associated with COVID-19 antigen testing uptake (χ^2^ = 10.16; *p* = 0.001). Specifically, bivariate logistic regression results indicated that women were significantly more likely to have been tested for COVID-19 antigens than were men (OR = 1.84; S.E. = 0.19; *p* = 0.002; 95% CI = 1.26, 2.69). Gender was also significantly associated with COVID-19 vaccination status (χ^2^ = 9.39; *p* = 0.002), with bivariate logistic regression results indicating that women were significantly more likely to have received at least one COVID-19 vaccine dose (OR = 1.79; S.E. = 0.19; *p* = 0.002; 95% CI = 1.23, 2.61). Age was also significantly associated with COVID-19 vaccination status (χ^2^ = 4.46; *p* = 0.035). Specifically, being aged 32 years or older was also significantly positively associated with the likelihood of having been vaccinated (OR = 1.48; S.E. = 0.19; *p* = 0.035; CI =1.03, 2.14), but was not associated with uptake of COVID-19 testing.

### 3.3. Associations between HIV Status, Hard Drug Use, and COVID-19 Service Uptake

Table 2 also presents Chi-square test results and logistic regression results for analyses examining associations of both HIV status and hard drug use with COVID-19 service uptake. Neither Chi-square tests nor unadjusted logistic regression detected an association between HIV status and likelihood of having been tested for COVID-19 antigens, nor did they detect an association between hard drug use and likelihood of having been tested for COVID-19 antigens. Similarly, neither of these analytic methods detected associations either between HIV status and COVID-19 vaccination uptake or between hard drug use and COVID-19 vaccination uptake. The adjusted logistic regression models we conducted as a sensitivity analysis that included HIV status (PLWH or not), hard drug use, gender, age, employment, and completion of high school as independent variables and either COVID-19 vaccination uptake or COVID-19 testing uptake found the same pattern of results as bivariate analyses: age was significantly associated only with vaccination uptake; gender was significantly associated with both vaccination and testing uptake; and neither HIV status nor hard drug use were associated with either service uptake measure.

### 3.4. Associations among HIV Status, Hard Drug Use, Vaccine Uptake, and COVID-19 IgG Antibody Status

Among the whole sample, Chi-square tests revealed significantly higher rates of vaccinated participants (N = 223, 85.2% of whom obtained vaccines over 6 months before the study) tested positive for COVID-19 IgG antibodies (χ^2^ = 87.10; *p* < 0.0005) compared to unvaccinated participants; and logistic regression found that vaccinated participants were significantly more likely to test positive for IgG antibodies (OR = 6.86; S.E. = 0.22; *p* < 0.0005; CI = 4.49, 10.49). This also held true among the subsample of PLWH (χ^2^ = 41.44; *p* < 0.0005; OR = 6.26; S.E. = 0.30; *p* < 0.0005; CI = 3.51, 11.20), as well as among only participants who were newly diagnosed with HIV (NDH; i.e., HIV-positive-unaware; χ^2^ = 12.13; *p* < 0.0005; OR = 8.40; S.E. = 0.64; *p* = 0.001; CI = 2.38, 29.66). Table 3 presents results from adjusted logistic regression models examining whether HIV status, NDH status, or hard drug use moderate the relationship between COVID-19 vaccination status and IgG antibody status. Adjusted logistic regression found that the interaction of a dummy variable for being NDH (or non-NDH) by vaccine status was not significantly associated with IgG antibody status net of the significant relationship between vaccine status and IgG antibody status. Similarly, adjusted logistic regression found that the interaction of a dummy variable for being HIV-positive by vaccine status was not significantly associated with IgG antibody status net of the significant relationship between vaccine status and IgG antibody status. In other words, neither NDH status nor HIV status significantly moderated the relationship between vaccine status and IgG antibody status. Likewise, another adjusted logistic regression model found that the interaction of hard drug use by vaccine status was not significantly associated with IgG antibody status net of the significant relationship between vaccine status and IgG antibody status, suggesting that hard drug use also does not moderate this relationship. The sensitivity analyses we conducted for these relationships additionally included age, employment, and completion of high school as covariates (but not gender, given the large proportion of variance it shares with vaccination status and concerns about multicollinearity). These sensitivity analyses found the same pattern of results: vaccination was significantly associated with IgG antibody status for all three models; and neither drug use, HIV status, nor the interactions of drug use or HIV status with vaccination was significantly associated with antibody status. A table of these findings is available in the online supplement (Table S1).

## 4. Discussion

The present study found that gender was significantly associated with uptake of both COVID-19 testing and COVID-19 vaccination, with larger proportions of women reporting having accessed both of these services. It also found that being older was significantly associated with a greater likelihood of having received a COVID-19 vaccine, but was not associated with COVID-19 testing uptake. Neither hard drug use nor HIV status was associated with COVID-19 testing or vaccination. While gender disparities in uptake of other kinds of healthcare are well-documented in this region (for example, in uptake of HIV testing and care [12,33,34,35]), there is relatively less previous literature to support interpretation of the relationship between age and COVID-19 vaccination in South Africa. Previous studies in South Africa, the United States, and elsewhere have found that younger adults are less likely to report that they would be willing to receive a COVID-19 vaccine (i.e., are more likely to report vaccine hesitancy [36,37,38]), and one study of vaccine uptake in South Africa conducted in September 2021—just after COVID-19 vaccination was made available to South African young adults (since it had previously only been available to adults over the age of 35)—found that younger people were less likely to have been vaccinated, but attributed this partially to the fact that they had had less time to access vaccination [39]. Since our study was conducted 1–1.5 years later, possible time for one to be able to access a vaccine is much less likely to be a confound, although timing of the vaccine’s initial availability vis-à-vis the time of most widespread concern about COVID-19 could still be an important factor. Other possible explanations for the relationship between age and COVID-19 vaccination are also plausible, however, given the findings from other studies that younger people are more likely to express vaccine hesitancy. For example, considering this relationship through the lens of the Health Belief Model [40], it may be that younger adults perceived the vaccine to be less beneficial to them (i.e., to have lower perceived benefits) since they were not considered as vulnerable to COVID-19 morbidity and mortality. Or, considering this relationship through the lens of Social Action Theory [41], there may have been norms among younger peer groups that shaped a lower degree of concern around COVID-19 and lower expectations of getting vaccinated, or even normative attitudes of disdain towards vaccination, whereas COVID-19 testing may have been standard practice (i.e., a norm in some settings) or even required as a condition of employment or educational activities for a period of time. These potential contributors to low COVID-19 vaccine uptake among young South Africans must be studied empirically. One study in South Africa found that COVID-19 vaccine hesitancy was associated with mistrust of the government and with anticipated COVID-19-related stigma [42], but these associations were not specific to younger adults. Future research should seek to unpack the reasons that younger adults were less likely to receive a COVID-19 vaccine, specifically, but were as likely as older adults to receive COVID-19 testing. Such research could contribute to understanding uptake of future vaccines for other infectious diseases, and is necessary to develop intervention and public health practice strategies to improve vaccination uptake among young people.

The present study also found that having been vaccinated for COVID-19 (over 6 months ago for 85% of our vaccinated participants) was significantly associated with COVID-19 IgG antibody positivity. This relationship held true among the subgroup of participants who were HIV-positive and among the subgroup of participants who were newly diagnosed with HIV (i.e., who had been HIV-positive-unaware at the time of COVID-19 IgG antibody testing). In other words, there was no evidence that HIV status or NDH status moderated the relationship between vaccine status and COVID-19 IgG antibody status. Though the detected association between vaccination history and IgG antibody positivity does not lend itself to any robust conclusions given that IgG antibody status is a reflection of a number of factors (including previous COVID-19 infection, which we did not measure), it is a potentially noteworthy finding that this relationship did not vary among the subgroup of participants who were HIV-positive-unaware. This may constitute preliminary evidence that post-vaccination immune responses did not significantly differ among people who were HIV-positive-unaware (i.e., people living with undiagnosed/untreated HIV); among PLWH who had already been diagnosed with HIV, most of whom were on ART; and among HIV-negative recipients of COVID-19 vaccines commonly administered to residents of the Msunduzi area. Though IgG antibody status could alternatively be a larger reflection of recent COVID-19 infection than of vaccination, the equivalence of relationships detected among each HIV status group is nonetheless an important finding, as it may suggest that people who are vaccinated and living with undiagnosed/untreated HIV are no more or less likely than other vaccinated people to have detectable COVID-19 IgG antibodies. This is an important addition to the previous literature on COVID-19 antibody response and HIV, as previous comparisons in antibody response between PLWH and people living without HIV have largely focused on comparing people living without HIV to PLWH receiving ART [6,7]. For example, one study in South Africa reported similar immune responses between people who did not have HIV infection and PLWH who were on ART, stably, for at least three months and who had viral loads of less than 1000 copies/mL [43]. The present study thus represents an important first exploratory step towards examining the relationship between COVID-19 vaccination and immune response among PLWH with a range of HIV control or progression and of engagement with ART (i.e., among participants who have a large degree of variability in their timing since diagnosis, many having been just diagnosed at the time of the study, and therefore in their opportunities to have had access to ART). Similar samples should be used to conduct future research with more robust measures of immune response.

### Limitations

The present study is limited by its cross-sectional design and as such, no causal inferences can be drawn. Future research must be conducted to understand the preliminary findings from our exploratory, descriptive analyses, including longitudinal analysis to establish temporal relationships between IgG antibody status and the potential predictors of interest, and additional qualitative or quantitative analysis to understand why young people and men were less likely to take up COVID-19-related services. Another major limitation of the present study is that we did not measure timing of COVID-19 testing among participants who reported having received such testing. Though we did ask whether participants who had had any COVID-19 antigen test had received a positive test result (ever), it would not be adequate to control for having ever had a positive COVID-19 antigen test in our analysis of COVID-19 antibody positivity, given that we cannot estimate the timing of the antigen test in relationship to vaccination or to the IgG antibody test we administered. As such, our analyses of COVID-19 IgG antibody positivity are limited to comparisons between groups based on HIV status and hard drug use. Future research should use multivariate models to examine whether HIV status or hard drug use moderates relationships among COVID-19 infection history, vaccination history, and IgG antibody status. Though a strength of the study is its inclusion of participants with both controlled HIV infection (i.e., participants who indicated that they have access to ART) and uncontrolled HIV infection (i.e., participants who were not diagnosed with HIV until the time of data collection), a limitation is that we were not able to measure any more specific indicators of HIV progression or control (e.g., viral load, length of time using ART). It is important for future research to include such indicators in future related research. Additional limitations of this study include reliance on self-report data to measure hard drug use and service access (since participant responses about both constructs could be affected by social desirability bias) and use of a somewhat socioculturally homogeneous sample, which is likely to limit generalizability of findings.

## 5. Conclusions

The present study found that neither HIV status nor hard drug use was associated with uptake of either COVID-19 antigen testing or COVID-19 vaccination, and that neither HIV status nor hard drug use moderated the significant relationship between COVID-19 vaccine status and COVID-19 IgG antibody status. Though more research is needed to support these preliminary, exploratory findings, this may suggest that two potentially vulnerable groups—people who use drugs and PLWH—were served as well as the general population by the COVID-19 response in KwaZulu-Natal. However, consistent with extant literature demonstrating that men have lower access than women to other kinds of healthcare in this geographic setting, the present study also found that there were similar gender-based disparities in uptake of COVID-19 testing and COVID-19 vaccination. Younger adults were also found to be less likely to have been vaccinated for COVID-19. This suggests that special care may need to be taken during future COVID-19 outbreaks or future new pandemics to implement interventions to improve access to related healthcare services among men and young adults in the Msunduzi area.

## Figures and Tables

**Table 1:** Descriptive statistics for sample of participants of a peer-recruitment-based HIV-testing intervention in KwaZulu-Natal (N = 470).

Variable	Category	Number	%
Gender	Man	287	61.1%
	Woman	183	38.9%
Age	18–31 years old	260	55.3%
	32 years or older	210	44.7%
Hard drug use in the last 6 months	Yes	51	10.9%
	No	419	89.1%
HIV status	PLWH (already aware)	180	38.3%
	NDH (previously unaware)	53	11.3%
	HIV-negative	237	50.4%
COVID-19 testing uptake	At least one test	247	52.6%
	Never	223	47.4%
COVID-19 vaccination status	At least one dose	223	47.4%
	None	247	52.6%
Most recent COVID-19 vaccine dose (N = 223)	More than 6 months ago	190	85.2%
	Within the last 6 months	33	14.8%
Number of COVID-19 vaccine doses (N = 223)	One	88	39.5%
	Two	133	59.6%
	Unsure	2	0.9%
COVID-19 IgG antibody test result	Positive	279	59.4%
	Negative	191	40.6%
Employed (part-time or full-time)	Yes	73	15.5%
	No	397	84.5%
Completed high school (standard 12 grades)	Yes	226	48.1%
	No	244	51.9%

**Table 2:** Results of Chi-square tests and unadjusted logistic regression models examining bivariate relationships of sociodemographic characteristics, HIV status, and hard drug use to uptake of COVID-19-related health services.

	COVID-19 Testing Uptake			COVID-19 Vaccination Status		
	Χ^2^	Odds Ratio †	95% C.I.	Χ^2^	Odds Ratio †	95% C.I.
Woman	10.16 **	1.84 **	1.26, 2.69	9.39 **	1.79 **	1.23, 2.61
32 years or older	1.10	1.22	0.84, 1.75	4.46 *	1.48 *	1.03, 2.14
Hard drug use	0.29	0.85	0.48, 1.53	1.55	0.69	0.38, 1.24
PLWH (or not)	0.08	1.05	0.73, 1.05	1.01	1.21	0.84, 1.73
NDH (or not)	0.69	0.78	0.44, 1.39	0.39	0.83	0.47, 1.48

* *p* < 0.05; ** *p* < 0.01; † Wald’s Z was used to evaluate the statistical significance of odds ratios for logistic regression.

**Table 3:** Results of adjusted logistic regression models testing whether hard drug use and/or HIV status moderate the relationship between COVID-19 vaccination status and COVID-19 IgG antibody status.

		Odds Ratio †	S.E.	95% C.I.
Model 1	PLWH	0.82	0.26	0.49, 1.37
	Vaccinated for COVID-19	8.01 ***	0.32	4.24, 15.11
	PLWH × Vaccination Interaction	0.78	0.44	0.33, 1.85
Model 2	NDH	0.63	0.42	0.28, 1.44
	Vaccinated for COVID-19	6.68 ***	0.23	4.25, 10.49
	NDH × Vaccination Interaction	1.26	0.68	0.33, 4.80
Model 3	Hard Drug Use	0.60	0.42	0.26, 1.35
	Vaccinated for COVID-19	6.11 ***	0.23	3.93, 9.52
	Hard Drug Use × Vaccination Interaction	3.60	0.87	0.65, 19.93

*** *p* < 0.0005. † Wald’s Z was used to evaluate the statistical significance of odds ratios for logistic regression.

## Data Availability

Given the small number of participants, the relationships of participants to each other (i.e., participants’ knowledge of other people’s participation in the study due to the use of peer recruitment), and the sensitive nature of HIV test results, data are not available for sharing at the suggestion of the research ethics committee, in order to protect the privacy of participants.

## Supplementary Materials

The following supporting information can be downloaded at: https://www.mdpi.com/article/10.3390/ijerph21111411/s1, Table S1: Sensitivity analysis: Results of adjusted logistic regression models testing whether hard drug use and/or HIV status moderate the relationship between COVID-19 vaccination status and COVID-19 IgG antibody status, net of sociodemographic covariates.

## Artificial-Intelligence-Assisted Technologies

No artificial-intelligence-assisted technologies were used in the production of this work.
