# Supplementary material for: Relationships Among COVID-19-Related Service Uptake, HIV Status, Drug Use, and COVID-19 Antibody Status Among HIV Testing Intervention Participants in KwaZulu-Natal, South Africa
Source: Int J Environ Res Public Health. 2024 Oct 25;21(11):1411. doi: 10.3390/ijerph21111411 (PMC11593625; doi:10.3390/ijerph21111411)
Supplement: Supplementary file 1 [file ijerph-21-01411-s001.zip › ijerph-3181779-supplementary.pdf]

Supplementary Materials

Table S1. Sensitivity Analysis: Results of adjusted logistic regression models testing whether hard drug use and/or HIV status moderate the relationship between COVID-19 vaccination status and COVID-19 IgG antibody status, net of sociodemographic covariates.

		**Odds Ratio †**	**S.E.**
Model 1	PLWH	0.77	0.27
	Vaccinated for COVID-19	7.89 ***	0.33
	PLWH x Vaccination Interaction	0.77	0.44
	Age	1.01	0.01
	Employed	1.19	0.30
	Completed High School	0.96	0.22
Model 2	NDH	0.64	0.42
	Vaccinated for COVID-19	6.60 ***	0.23
	NDH x Vaccination Interaction	1.24	0.69
	Age	1.00	0.01
	Employed	1.17	0.30
	Completed High School	1.00	0.22
Model 3	Hard Drug Use	0.61	0.42
	Vaccinated for COVID-19	6.03 ***	0.23
	Hard Drug Use x Vaccination Interaction	3.63	0.87
	Age	1.00	0.01
	Employed	1.18	0.30
	Completed High School	1.01	0.22

* $p < 0.05$; ** $p < 0.01$; *** $p < 0.0005$; † Wald's Z was used to evaluate the statistical significance of odds ratios for logistic regression.
